# A Computational Framework to Identify Metabolic Engineering Strategies for the Co-Production of Metabolites

**DOI:** 10.3389/fbioe.2021.779405

**Published:** 2022-01-07

**Authors:** Lavanya Raajaraam, Karthik Raman

**Affiliations:** ^1^ Department of Biotechnology, Bhupat and Jyoti Mehta School of Biosciences, Indian Institute of Technology (IIT) Madras, Chennai, India; ^2^ Centre for Integrative Biology and Systems mEdicine (IBSE), IIT Madras, Chennai, India; ^3^ Robert Bosch Centre for Data Science and Artificial Intelligence (RBCDSAI), IIT Madras, Chennai, India

**Keywords:** metabolic modelling, genome-scale models, bioproduction, concomitant production, co-synthesis, constraint-based modelling

## Abstract

Microbial production of chemicals is a more sustainable alternative to traditional chemical processes. However, the shift to bioprocess is usually accompanied by a drop in economic feasibility. Co-production of more than one chemical can improve the economy of bioprocesses, enhance carbon utilization and also ensure better exploitation of resources. While a number of tools exist for *in silico* metabolic engineering, there is a dearth of computational tools that can co-optimize the production of multiple metabolites. In this work, we propose co-FSEOF (co-production using Flux Scanning based on Enforced Objective Flux), an algorithm designed to identify intervention strategies to co-optimize the production of a set of metabolites. Co-FSEOF can be used to identify all pairs of products that can be co-optimized with ease using a single intervention. Beyond this, it can also identify higher-order intervention strategies for a given set of metabolites. We have employed this tool on the genome-scale metabolic models of *Escherichia coli* and *Saccharomyces cerevisiae*, and identified intervention targets that can co-optimize the production of pairs of metabolites under both aerobic and anaerobic conditions. Anaerobic conditions were found to support the co-production of a higher number of metabolites when compared to aerobic conditions in both organisms. The proposed computational framework will enhance the ease of study of metabolite co-production and thereby aid the design of better bioprocesses.

## 1 Introduction

Recent years have seen several advances in the usage of bioprocessing to produce a wide range of chemicals (Erickson et al., 2012). Microorganisms can produce diverse and complex products from simple carbon sources. Nevertheless, there are many challenges in designing economically feasible bioprocesses. The advancements in synthetic biology have enabled the metabolic engineering of organisms to improve yield and productivity (Yadav et al., 2019). Various computational strain design algorithms have been developed to identify the genetic manipulations required to over-produce a single product (Burgard et al., 2003; Rocha et al., 2008; Yang et al., 2011). Despite the increase in yield achieved through such rational strain design, the bioprocesses are unable to compete with the traditional chemical processes in many cases (Cai and Bennett, 2011). This is due to two main reasons: 1) the cost of raw materials and 2) the maximum yield achievable for a given product in a given organism and environment is limited by the number of genetic manipulations that can be successfully implemented in a single strain (Silva et al., 2012). The former issue can be reduced by using agricultural waste as feedstock instead of a synthetic nutrient medium. The latter can be overcome by co-producing multiple products in the same bioprocess (da Silva et al., 2014).

Co-production equips us to exploit the system in a better fashion and produce more valuable products from the same raw materials. A high-value, low-volume chemical can be co-produced with a low-value, high-volume product in order to increase the economic feasibility, as in the case of riboflavin and butanol, respectively (Cai and Bennett, 2011). Co-production is essential when a cocktail of metabolites need to be produced together, rather than a single metabolite, as in the case of biofuels and fatty acids (Xin et al., 2018). A mixture of different alcohols or fatty acids of varying chain length need to be co-optimized in such cases. It can also balance carbon metabolism, as in the case of uridine, and acetoin (Fan et al., 2018). High carbon inflow towards uridine causes excess production of acetate, which hampers the growth of the organism. Conversion of acetate to acetoin prevents over-acidification of the nutrient medium and thereby improves growth and uridine production. There are many studies that have successfully achieved co-production of a variety of products with/without genetic manipulation of the organisms. Polyhydroxyalkanoates are a common class of metabolites that are co-produced with other metabolites (Li et al., 2017; Kumar and Kim, 2018; Yadav et al., 2021). Butanol and hydrogen have been co-produced in *Clostridium beijerinckii* (Zhang et al., 2021), and ethanol and xylitol have been co-produced in *Candida tropicalis* (Raj and Krishnan, 2020; de Souza Queiroz et al., 2021). The carbon source, nutrient medium, pH etc., are optimized in such cases to improve the yield of metabolites. Metabolic engineering can further expand the number of products that are co-produced and also improve their yield significantly. Multiple metabolites like ethanol, isopropanol, butanol and 2,3- butanediol have been co-produced by optimizing the acetone-butanol-ethanol (ABE) fermentation pathway in *Clostridium acetobutylicum* (Collas et al., 2012). Nisin and 3-phenyllactic acid, two antimicrobial agents, have been co-produced in *Lactococcus lactis* through genetic manipulation (Julien-Laferrière et al., 2016). Non-native metabolites can also be co-produced with other metabolites, as in the case of butanol and riboflavin, by engineering the heterologous pathway in *C. acetobutylicum* (Cai and Bennett, 2011).

Although many strain design algorithms have been successfully employed for metabolically engineering organisms to optimize a single product (Pharkya et al., 2003; Kumelj et al., 2019), few studies have applied it for co-production. The studies listed above only use existing literature and readily apparent deletion targets to achieve co-production. This limits the robustness of the bioprocesses that are designed. There is a lack of algorithms that can be easily applied to study co-production. In this study, we have extended the Flux Scanning based on Enforced Objective Flux (FSEOF) (Choi et al., 2010) algorithm to study co-production. Further, while deletion targets can be obtained for metabolites independently using existing algorithms like OptKnock (Burgard et al., 2003), OptGene (Rocha et al., 2008), there are very few algorithms that can identify amplification targets (Ranganathan et al., 2010). In order to identify amplification targets in addition to knock-out targets for co-optimizing a set of metabolites, we propose a new methodology, co-FSEOF, adapting the FSEOF algorithm. Co-FSEOF has a simple computational framework that can be easily modified, and it also provides the entire set of potential intervention strategies in a single run while many algorithms are sequential, returning one intervention target per run. The utility of the potential intervention strategies obtained was further assessed using Flux Variability Analysis (FVA). We applied co-FSEOF to evaluate all possible pairs of secretory metabolites in *Escherichia coli* and *Saccharomyces cerevisiae.* The different pairs of metabolites that can be co-produced through a single reaction deletion or amplification were obtained. This analysis helps us choose favorable pairs of metabolites for which higher-order intervention strategies can be obtained. We have demonstrated this by identifying the amplification targets, knock-out targets and mixed intervention strategies of size up to three to co-optimize the production of isobutanol and succinic acid in *S. cerevisiae.* Higher-order intervention strategies were able to achieve better yield with very little reduction in growth rate. Overall, our analyses provide an overall picture of the biosynthetic capabilities of an organism, particularly highlighting key interdependencies in metabolism.

## 2 Methods

### 2.1 Flux Balance Analysis

**FIGURE 1:**
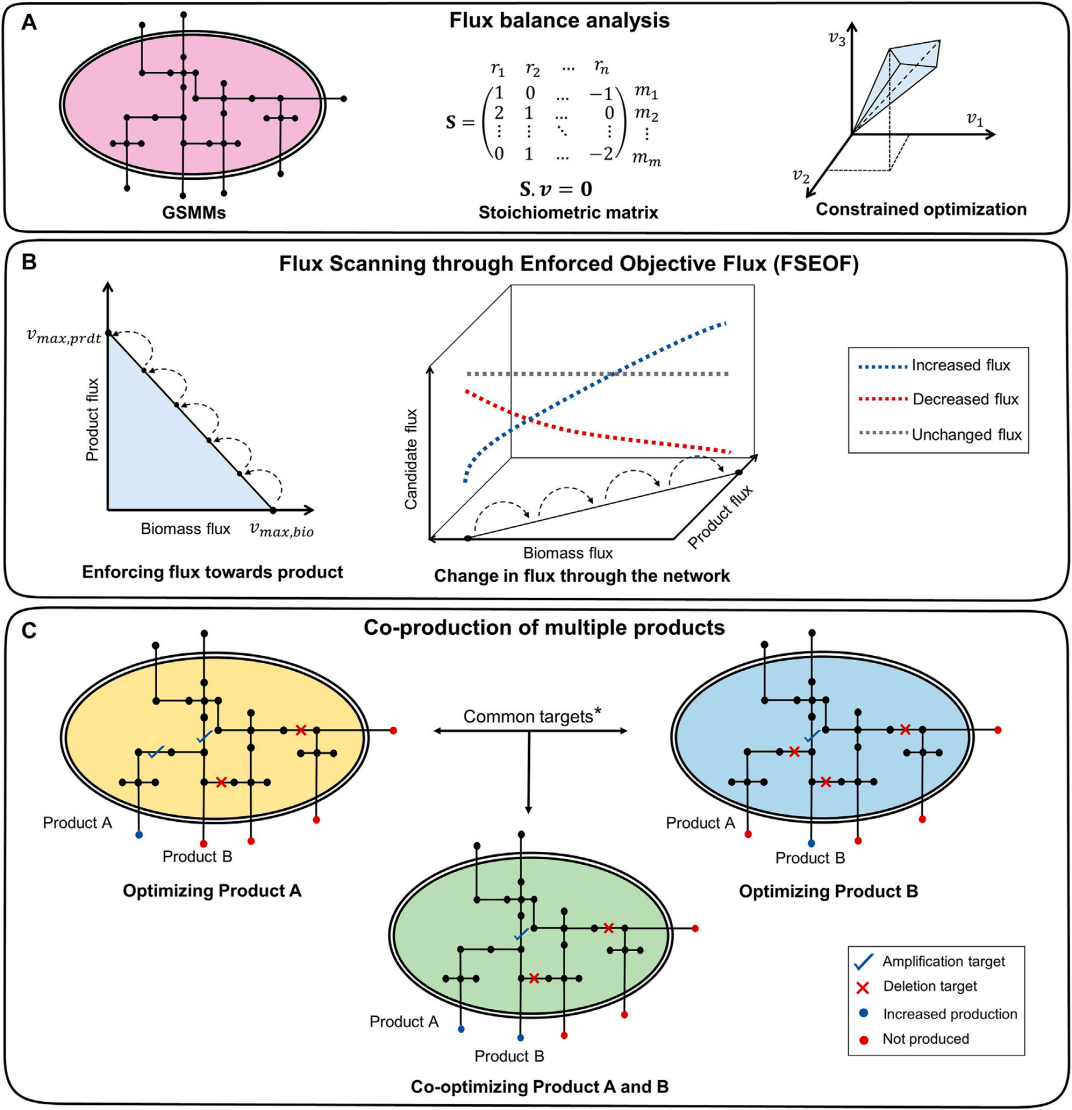
Framework of co-FSEOF. **(A)** The GSMM is represented as a stoichiometric matrix, which is used for FBA. **(B)** The flux through the product is increased in steps, and flux changes through all other reactions are studied. The reactions that have increased flux with an increase in product flux are potential amplification targets. The reactions that have decreased flux are potential deletion targets, while those with unchanged or oscillatory fluxes are excluded. **(C)** The targets common to products A and B are the potential targets for co-optimization. *The union of all potential targets for products A and B is used for higher-order intervention strategies.

FBA is a widely used steady-state constraint-based modelling approach to predict the metabolic capabilities of a variety of organisms (Varma and Palsson, 1994; Kauffman et al., 2003; Orth et al., 2010). The metabolic network of an organism, which comprises all reactions known to occur in the organism, is represented as a stoichiometric matrix S , of size m×n , where m is the number of metabolites, and n is the number of reactions (Figure 1A). The entries in the j th column of S represent the stoichiometric coefficients of the metabolites that participate in the j th reaction. The minimum and maximum values of flux that any reaction can assume are constrained by the lower and upper bounds, respectively. The flux through a reaction under a given set of conditions, at steady-state, is calculated by solving a linear programming (LP) problem. The LP problem is formulated as:

$$\max_{v}\;\;\;\;\;\;\;\;\;\;c^{T}v\;$$

subject to S.v=0

$$\mathrm{while}\;v_{l,i}\leq v_{i}\leq v_{u,i},\forall i\in \left[1,\;n\right]$$

where c is a vector of weights denoting the contribution of each of the n reactions to the objective function, $v\;\epsilon \;R^{n}$ is the vector of metabolic fluxes, $v_{l}$ and $v_{u}$ are vectors representing the lower and upper bounds for the reaction fluxes, respectively (Orth et al., 2010).

### 2.2 Flux Variability Analysis

FVA is used to identify the range of fluxes of each reaction that still satisfy the constraints, where two optimization problems are solved for each flux $v_{i}\;$ of interest.

$$\;v_{j,max}/v_{j,min}=max_{v}/min_{v}\cdot v_{j}$$

s.t. S.v=0

$$\mathrm{while}\;v_{l,i}\leq v_{i}\leq v_{u,i}\;,\;\forall i\in \left[1,\;n\right]$$

where $v\;\epsilon \;R^{n}$ is the vector of metabolic fluxes, $v_{j,max}$ and $v_{j,min}$ are the maximum and minimum values of fluxes, respectively for each reaction flux $v_{j}$ (Gudmundsson and Thiele, 2010).

### 2.3 Flux Scanning Based on Enforced Objective Flux

FSEOF (Choi et al., 2010) is a method used to identify potential reaction deletion and amplification targets in metabolic networks by observing the change in the reaction fluxes when the system moves from the wild-type flux of the target product to the theoretical maximum flux of the product (Figure 1B). The maximum biomass $v_{\text{max},bio}$ and maximum product $v_{\text{max},prdt}$ fluxes are obtained by performing FBA with the biomass reaction and the exchange reaction of the product as the objective, respectively. The flux of the target reaction, $v_{prdt}^{\;}$ is pinned to $x\%\;of\;v_{\text{max},prdt}\;\left(x=0\to 100\right).$ The change in the flux of a reaction, $v_{j}$ , is studied as the product flux, $v_{prdt}^{\;}$ is increased, and it is classified as a potential deletion or amplification target based on the decrease or increase in its flux, respectively. The reactions that undergo no change or oscillations in the fluxes are discarded from the set of potential intervention strategies. The set of potential intervention strategies obtained are assessed by simulating each intervention and performing FVA on the mutant.

### 2.4 Co-FSEOF: Co-Optimization of Metabolites

**FIGURE 2:**
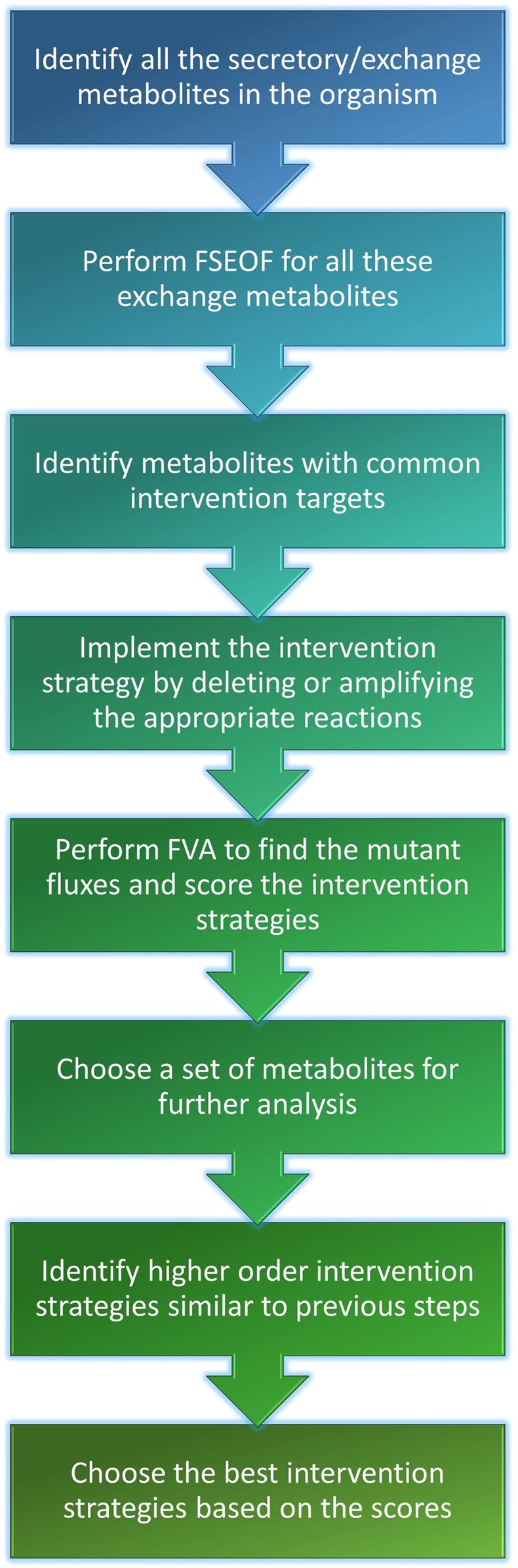
Workflow of co-FSEOF. The figure illustrates succinctly, the key steps of the algorithm.

The Genome-Scale Metabolic Models (GSMMs) of *E. coli* iML1515 and *S. cerevisiae* iMM904 were obtained from the BiGG models database (http://bigg.ucsd.edu/) (Raj and Krishnan, 2020). The simulations were done with the following constraints on uptakes: −10 mmol/gDW/h glucose and −2 mmol/gDW/h oxygen for aerobic conditions and −10 mmol/gDW/h glucose and zero oxygen uptake for anaerobic conditions. Exchange and transport reactions were removed from the search space for FSEOF to increase the relevance of the results and to reduce the computational time. The potential intervention strategies for all secretory metabolites (metabolites that can be secreted into the medium) in the organism were obtained using FSEOF as described in Section 2.3. All possible pairs of secretory metabolites were examined for co-production by identifying their common intervention strategies which were obtained through FSEOF (Figure 1C). These common deletion or amplification targets constitute the potential intervention strategies for a set of metabolites. The implementation steps of the algorithm are summarized in Figure 2.

The reliability of the potential intervention targets is analyzed by comparing the flux values of biomass and product in the mutant with those of the wild-type organism. The mutant model is obtained by deleting the reaction in case of knock-out targets or by fixing the flux bounds of the amplification target to its theoretical maximum. FVA is performed on this mutant model with biomass as objective to obtain the range of flux values for the products and biomass. Any reaction with more than a 5% increase in the maximum product flux and less than 75% decrease in biomass flux is considered a promising intervention strategy. FVA was performed using fastFVA to reduce the computational time (Gudmundsson and Thiele, 2010).

To obtain higher-order intervention strategies, the potential targets obtained earlier for a given set of metabolites were combined, and all possible combinations of intervention strategies of a certain size (up to three) were evaluated using FVA. The score for each product i (total number of products, n ) and the overall score are calculated as

$$Score_{i}=\;\frac{\left(v_{prdt_{i},mut}-v_{prdt_{i},wt}\right)}{\left(v_{bio,wt}-v_{bio,mut}\right)}$$

$$\mathrm{Overall}\;\mathrm{Score}=\overset{n}{\underset{i=1}{\sum }}\left(\mathrm{Scor}e_{i}\right)$$

where $v_{prdt_{i},mut}$ and $v_{prdt_{i},wt}$ are the mutant and wild-type maximum fluxes of the exchange reaction of the product i , and $v_{bio,mut}$ and $v_{bio,wt}$ are the mutant and wild-type fluxes of the biomass reaction. $Score_{i}$ denotes the score for the individual product while overall score denotes the cumulative score for the set of metabolites. All simulations were performed in MATLAB R2018a (MathWorks Inc., United States) using the COBRA Toolbox v3.0 (Heirendt et al., 2019) and IBM ILOG CPLEX 12.8 as the linear programming solver.

## 3 Results

Metabolic engineering strategies for the co-production of all pairs of secreted metabolites in *E. coli* and *S. cerevisiae* were obtained using co-FSEOF as described in Section 2.4. We identified the intervention strategies required to optimize the co-production of metabolites in both aerobic and anaerobic conditions. Anaerobic conditions favor the co-production of more pairs of metabolites when compared to aerobic conditions. The intervention strategy for each pair of metabolites is scored as in Section 2.4. The best intervention strategy can be chosen using the overall score . In cases where one metabolite might be favored over the others, the individual scores, $Score_{i}$ can be used to choose the best intervention strategies. Some of the intervention strategies obtained have been successfully verified through experimental studies in literature. This shows the credibility of the intervention strategies obtained. We discuss a few of the industrially significant metabolites and their intervention strategies, along with supporting literature. We also propose many other intervention strategies, which form a ready short-list for experimental validation. We were able to identify other hitherto unexplored intervention strategies, which may be better alternatives to those in existing literature, further demonstrating the utility of the algorithm.

### 3.1 Co-Production in *Escherichia coli*

*E. coli* is one of the well-studied model organisms and has high-quality GSMMs available. The latest GSMM, *i*ML1515 (Monk et al., 2017), was used in this study, and the co-production of 337 secretory metabolites was studied in both aerobic and anaerobic conditions.

#### 3.1.1 Aerobic Fermentation

Co-production of all pairs of metabolites was studied in *E. coli*, using co-FSEOF and FVA as described in Section 2.3, 2.4. Out of C2337 pairs of secretory metabolites, only 237 could be successfully overproduced through deletion or amplification of a single reaction. The intervention strategies for a few industrially significant pairs of metabolites are listed in Table 1. One of the important pairs of metabolites that can be easily co-produced is L-lysine, a food additive and drug additive and cadaverine, which is essential for polyamide production. co-FSEOF was able to identify several reactions from the diaminopimelate pathway (DAP), which can be over-expressed to co-produce L-lysine and cadaverine. An experimental study by Xu et al. (2019) demonstrates the effect of engineering the DAP pathway in *E. coli* for the production of L-lysine. This indicates the reliability of the results obtained through our computational approach. Another significant result is the co-production of succinate and ethanol through the amplification of glyceraldehyde-3-phosphate dehydrogenase. Other studies have also successfully co-produced ethanol and succinate by other genetic manipulations (Liang et al., 2019).

**TABLE 1:** Intervention strategies for co-production of pairs of metabolites in *E. coli* under aerobic conditions.

#	Product A	WT flux A	Product B	WT flux B	Intervention	Mutant product flux A	Mutant product flux B	Mutant biomass flux	Score A	Score B	Score A + B	KO/Amp
1	L-lysine	*	1,5-Diamino pentane	*	Diaminopimelate decarboxylase	5.81	5.81	0.22	8.84	8.84	17.68	Amp
					Diaminopimelate epimerase	5.81	5.81	0.22	8.84	8.84	17.68	Amp
					Dihydrodipicolinate reductase	5.81	5.81	0.22	8.84	8.84	17.68	Amp
					Dihydrodipicolinate synthase	5.81	5.81	0.22	8.84	8.84	17.68	Amp
					Succinyl-diaminopimelate desuccinylase	5.81	5.81	0.22	8.84	8.84	17.68	Amp
					Tetrahydrodipicolinate succinylase	5.81	5.81	0.22	8.84	8.84	17.68	Amp
2	Succinate	*	Ethanol	*	Glyceraldehyde-3-phosphate dehydrogenase	12.86	15.84	0.22	19.56	24.10	43.67	Amp
3	Spermidine	*	5-Methylthio-D-ribose	*	Adenosylmethionine decarboxylase	2.01	2.01	0.22	3.06	3.06	6.12	Amp
					Methylthioadenosine nucleosidase	2.01	2.01	0.22	3.06	3.06	6.12	Amp
					Spermidine synthase	2.01	2.01	0.22	3.06	3.06	6.12	Amp
4	Xanthine	*	D-Lactate	*	Glyceraldehyde-3-phosphate dehydrogenase	8.90	15.84	0.22	13.55	24.10	37.65	Amp
5	Glycine	*	L-Asparagine	*	Glyceraldehyde-3-phosphate dehydrogenase	20.91	11.82	0.22	31.80	17.99	49.79	Amp
6	Fe-enterobactin	*	Enterobactin	*	2,3-Dihydro-2,3-dihydroxybenzoate dehydrogenase	1.29	1.29	0.22	1.97	1.97	3.93	Amp
					Isochorismatase	1.29	1.29	0.22	1.97	1.97	3.93	Amp
7	Pyruvate	*	L-Asparagine	*	Glyceraldehyde-3-phosphate dehydrogenase	18.01	11.82	0.22	27.39	17.99	45.38	Amp

WT, wild type; *, less than 10^−5^ mmol/gDW/h.

#### 3.1.2 Anaerobic Fermentation

Anaerobic conditions support the co-production of more metabolites when compared to aerobic conditions. More than 1,000 pairs of metabolites can be co-produced, out of which few are listed in Table 2. L-lysine and cadaverine can be co-produced under anaerobic conditions too. But the maximum flux achievable is lower when compared to aerobic conditions. The yield of metabolites like acetate, formate, and hexanoate can be co-optimized by deleting acetaldehyde dehydrogenase or alcohol dehydrogenase. We also found that succinate and lactate can be co-produced by the knock-out of pyruvate formate lyase. The effect of deletion of *pflB* gene encoding pyruvate formate lyase has been experimentally verified in *E. coli* for succinate production (Zhang et al., 2009) and lactate production (Utrilla et al., 2009) through separate studies. This shows that there are multiple co-production strategies available in the existing literature that can be easily utilized to design an efficient process.

**TABLE 2:** Intervention strategies for co-production of pairs of metabolites in *E. coli* under anaerobic conditions.

#	Product A	WT flux A	Product B	WT flux B	Intervention	Mutant product flux A	Mutant product flux B	Mutant biomass flux	Score A	Score B	Score A + B	KO/Amp
1	Acetate	8.83	Formate	18.22	Acetaldehyde dehydrogenase	18.18	36.79	0.12	237.95	472.31	710.26	KO
					Alcohol dehydrogenase	18.18	36.79	0.12	237.95	472.31	710.26	KO
2	Succinate	0.05	D-Lactate	3.76x10^−4^	Glyceraldehyde-3-phosphate dehydrogenase	14.98	19.41	0.04	126.78	164.92	291.71	Amp
					Acetaldehyde dehydrogenase	9.07	18.12	0.12	229.50	461.04	690.55	KO
					Alcohol dehydrogenase	9.07	18.12	0.12	229.50	461.04	690.55	KO
					Pyruvate formate lyase	0.49	17.76	0.12	10.57	426.65	437.22	KO
3	L-Alanine	*	Xanthine	*	Glyceraldehyde-3-phosphate dehydrogenase	13.56	0.97	0.04	115.18	8.23	123.41	Amp
4	Spermidine	*	5-Methylthio-D-ribose	*	Adenosylmethionine decarboxylase	0.64	0.64	0.04	5.46	5.46	10.93	Amp
					Methylthioadenosine nucleosidase	0.64	0.64	0.04	5.46	5.46	10.93	Amp
					Spermidine synthase	0.64	0.64	0.04	5.46	5.46	10.93	Amp
5	L-Aspartate	*	L-Glutamate	*	Glyceraldehyde-3-phosphate dehydrogenase	6.03	4.52	0.04	51.19	38.39	89.59	Amp
6	L-Lysine	*	1,5-Diamino pentane	*	Diaminopimelate decarboxylase	2.72	2.72	0.04	23.04	23.04	46.07	Amp
					Diaminopimelate epimerase	2.72	2.72	0.04	23.04	23.04	46.07	Amp
					Dihydrodipicolinate synthase	2.72	2.72	0.04	23.04	23.04	46.07	Amp
					Succinyl-diaminopimelate desuccinylase	2.72	2.72	0.04	23.04	23.04	46.07	Amp
					Tetrahydrodipicolinate succinylase	2.72	2.72	0.04	23.04	23.04	46.07	Amp
7	Acetate	8.83	Hexanoate	*	Acetaldehyde dehydrogenase	18.18	4.53	0.12	237.95	115.26	353.21	KO
					Alcohol dehydrogenase	18.18	4.53	0.12	237.95	115.26	353.21	KO

WT, wild type; *, less than 10^−5^ mmol/gDW/h.

### 3.2 Co-Production in *Saccharomyces cerevisiae*

Another industrially relevant and well-studied model organism is *S. cerevisiae.* Though heterologous pathways have not been analyzed in this study, one can easily modify the GSMM and apply co-FSEOF to identify co-production strategies for heterologous metabolites. Since *S. cerevisiae* is a better candidate for recombinant protein production, it is essential to study co-production in yeast (Bill, 2014). It can also produce more complex metabolites when compared to *E. coli* and is, therefore, a favorable candidate for bio-production. The latest GSMM iMM904 (Mo et al., 2009) was used, and the ability to optimize the co-production of 164 secretory metabolites was studied in both aerobic and anaerobic conditions.

#### 3.2.1 Aerobic Fermentation

We found that many industrially important metabolites like ethanol and L-alanine, and 4-aminobutanoate and L-serine can be co-produced in *S. cerevisiae* under aerobic conditions. We were also able to co-optimize isobutyl alcohol and 2-methyl propanal, which are long-chain alcohols that are used as biofuels. The deletion of pyruvate dehydrogenase increases the production of pyruvate and acetate, as shown in Table 3. Although the deletion of pyruvate dehydrogenase has not been experimentally verified as yet, a similar study has been carried out in *E. coli* (Moxley and Eiteman, 2021). In this study, it has been shown that the deletion of the genes encoding pyruvate dehydrogenase improves pyruvate production (Moxley and Eiteman, 2021). In addition to pyruvate dehydrogenase, co-FSEOF was able to identify several other amplification targets, which can also improve the production of pyruvate and acetate.

**TABLE 3:** Intervention strategies for co-production of pairs of metabolites in *S. cerevisiae* under aerobic conditions.

#	Product A	WT flux A	Product B	WT flux B	Intervention	Mutant product flux A	Mutant product flux B	Mutant biomass flux	Score A	Score B	Score A + B	KO/Amp
1	Ethanol	15.81	L-Alanine	1.69x10^−4^	Sedoheptulose 1,7-bisphosphate D-glyceraldehyde-3-phosphate-lyase	18.45	0.15	0.07	12.22	0.71	12.93	Amp
					Phosphofructokinase (s7p)	18.45	0.15	0.07	12.22	0.71	12.93	Amp
2	Acetate	3.55×10^−3^	2,3-Butanediol	3.21x10^−4^	Pyruvate dehydrogenase	1.48	0.25	0.28	230.94	38.48	269.42	KO
					Enolase	10.71	10.27	0.07	49.61	47.61	97.22	Amp
					Fructose-bisphosphate aldolase	10.70	10.27	0.07	49.62	47.64	97.26	Amp
					Glyceraldehyde-3-phosphate dehydrogenase	10.71	10.27	0.07	49.61	47.61	97.22	Amp
					Triose-phosphate isomerase	10.70	10.27	0.07	49.62	47.64	97.26	Amp
3	Isobutyl alcohol	*	Succinate	*	Pyruvate decarboxylase	7.63	5.45	0.19	79.29	56.66	135.95	KO
					Enolase	9.56	12.97	0.07	44.30	60.10	104.40	Amp
					Glyceraldehyde-3-phosphate dehydrogenase	9.56	12.97	0.07	44.30	60.13	104.43	Amp
					Triose-phosphate isomerase	9.56	12.97	0.07	44.33	60.17	104.50	Amp
4	2-Methylpropanal	*	Isobutyl alcohol	*	3-Methyl-2-oxobutanoate decarboxylase	6.50	9.36	0.07	30.14	43.39	73.54	Amp
					Acetolactate synthase mitochondrial	6.50	9.36	0.07	30.14	43.40	73.54	Amp
					Dihydroxy acid dehydratase 2,3-dihydroxy-3-methylbutanoate mitochondrial	6.50	9.36	0.07	30.14	43.40	73.54	Amp
					Enolase	8.95	9.56	0.07	41.47	44.30	85.77	Amp
					Glyceraldehyde-3-phosphate dehydrogenase	8.95	9.56	0.07	41.47	44.30	85.77	Amp
					Acetohydroxy acid isomeroreductase mitochondrial	6.50	9.36	0.07	30.14	43.40	73.54	Amp
					Triose-phosphate isomerase	8.94	9.56	0.07	41.49	44.33	85.82	Amp
5	L-Glutamate	*	2-Oxoglutarate	*	Citrate synthase	2.93	1.95	0.07	13.58	9.05	22.63	Amp
					Enolase	4.50	4.04	0.07	20.85	18.73	39.58	Amp
					Fructose-bisphosphate aldolase	4.50	4.04	0.07	20.86	18.73	39.60	Amp
					Glyceraldehyde-3-phosphate dehydrogenase	4.50	4.04	0.07	20.85	18.73	39.58	Amp
					Isocitrate dehydrogenase	2.99	2.00	0.07	13.87	9.25	23.12	Amp
					Triose-phosphate isomerase	4.50	4.04	0.07	20.86	18.73	39.60	Amp
6	Acetate	3.55×10^−3^	Pyruvate	3.24x10^−4^	Pyruvate dehydrogenase	1.48	0.25	0.28	230.94	38.48	269.42	KO
					Aspartate-semialdehyde dehydrogenase	8.79	11.85	0.07	41.14	55.49	96.63	Amp
					Enolase	10.71	14.42	0.07	49.61	66.83	116.44	Amp
					Fructose-bisphosphate aldolase	10.70	14.41	0.07	49.62	66.85	116.47	Amp
					Glyceraldehyde-3-phosphate dehydrogenase	10.71	14.42	0.07	49.61	66.83	116.44	Amp
					Triose-phosphate isomerase	10.70	14.41	0.07	49.62	66.85	116.47	Amp
7	4-Aminobutanoate	*	L-Serine	*	Glyceraldehyde-3-phosphate dehydrogenase	4.77	6.98	0.07	22.10	32.34	54.44	Amp
					Triose-phosphate isomerase	4.77	6.97	0.07	22.11	32.35	54.45	Amp
8	L-Alanine	1.69x10^−4^	L-Cysteine	*	Glucose-6-phosphate dehydrogenase	10.60	1.20	0.07	49.11	5.56	54.67	Amp
					Phosphogluconate dehydrogenase	10.60	1.20	0.07	49.11	5.56	54.67	Amp
					6-phosphogluconolactonase	10.60	1.20	0.07	49.11	5.56	54.67	Amp
					Ribulose-5-phosphate-3-epimerase	10.60	1.20	0.07	49.11	5.56	54.67	Amp
					Transketolase	10.60	1.20	0.07	49.12	5.56	54.68	Amp
					Ribose-5-phosphate isomerase	18.16	2.21	0.08	85.44	10.40	95.84	Amp
9	sn-Glycero-3-phosphocholine	*	L-Methionine	*	Methionine synthase	0.02	2.19	0.07	0.11	10.17	10.27	Amp
					5,10-Methylene-tetrahydrofolate reductase	0.02	2.19	0.07	0.11	10.17	10.27	Amp
					Ribose-5-phosphate isomerase	0.66	1.71	0.08	3.11	8.03	11.14	Amp
10	2-Methylbutyl acetate	*	2-Methyl-1-butanol	*	Ribose-5-phosphate isomerase	3.00	4.42	0.08	14.13	20.78	34.92	Amp

WT, wild type; *, less than 10^−5^ mmol/gDW/h.

#### 3.2.2 Anaerobic Fermentation

As in the case of *E. coli,* anaerobic fermentation enables the co-production of more pairs of metabolites in *S. cerevisiae* when compared to aerobic fermentation. 2-methyl-1-butanol, which is an important solvent used in the manufacture of pesticides and paints and isobutyl alcohol, which is a biofuel, can be co-produced by the amplification of malic enzyme, as shown in Table 4. Formate, which is used in dyeing and printing, can be co-produced with spermidine, a metabolite increasingly studied for its anti-ageing properties (Minois, 2014), through the amplification of a number of reactions. These strategies not only include readily apparent reactions that are involved in spermidine synthesis like spermidine synthase and adenosylmethionine decarboxylase but also provide some non-intuitive strategies like the amplification of aspartate transaminase or 2-keto-4-methylthiobutyrate transaminase. We also found that the deletion of pyruvate decarboxylase improves the production of succinate, isobutyl alcohol and pyruvate. The effect of deletion of pyruvate decarboxylase has been studied in *S. cerevisiae,* and the improvement in the production of pyruvate (van Maris et al., 2004) and succinate (Zahoor et al., 2019) has been verified experimentally in separate studies in literature.

**TABLE 4:** Intervention strategies for co-production of pairs of metabolites in *S. cerevisiae* under anaerobic conditions.

#	Product A	WT flux A	Product B	WT flux B	Intervention	Mutant product flux A	Mutant product flux B	Mutant biomass flux	Score A	Score B	Score A + B	KO/Amp
1	2-Methyl-1-butanol	*	Isobutyl alcohol	*	Malic enzyme NADP mitochondrial	0.03	9.64	0.05	0.20	61.30	61.51	Amp
2	Isobutyl alcohol	*	Pyruvate	*	Pyruvate decarboxylase	8.73	5.66	0.11	88.74	57.57	146.31	KO
					Enolase	9.68	9.97	0.05	61.59	63.45	125.03	Amp
					Fructose-bisphosphate aldolase	9.68	9.96	0.05	61.67	63.47	125.14	Amp
					Glyceraldehyde-3-phosphate dehydrogenase	9.68	9.97	0.05	61.60	63.45	125.05	Amp
					Triose-phosphate isomerase	9.68	9.96	0.05	61.66	63.47	125.13	Amp
3	Formate	6.26x10^−4^	Spermidine	*	Adenosylmethionine decarboxylase	1.22	1.22	0.05	7.76	7.76	15.52	Amp
					Aspartate transaminase	1.21	1.21	0.05	7.75	7.76	15.51	Amp
					2,3-Diketo-5-methylthio-1-phosphopentane degradation	1.22	1.22	0.05	7.76	7.76	15.52	Amp
					5-Methylthio-5-deoxy-D-ribulose-1-phosphate dehydratase	1.22	1.22	0.05	7.76	7.76	15.52	Amp
					5-Methylthioadenosine phosphorylase	1.22	1.22	0.05	7.76	7.76	15.52	Amp
					5-Methylthioribose-1-phosphate isomerase	1.22	1.22	0.05	7.76	7.76	15.52	Amp
					Spermidine synthase	1.22	1.22	0.05	7.76	7.76	15.52	Amp
					2-Keto-4-methylthiobutyrate transamination	1.22	1.22	0.05	7.76	7.76	15.52	Amp
4	4-Amino butanoate	*	Isobutyl acetate	*	Enolase	2.84	2.91	0.05	18.04	18.50	36.53	Amp
					Fructose-bisphosphate aldolase	2.84	2.90	0.05	18.11	18.50	36.61	Amp
					Glyceraldehyde-3-phosphate dehydrogenase	2.85	2.91	0.05	18.11	18.50	36.61	Amp
					Triose-phosphate isomerase	2.84	2.90	0.05	18.11	18.50	36.61	Amp
5	2-Methyl-1-butanol	*	Glycine	*	Aspartate kinase	0.05	4.55	0.05	0.32	28.95	29.26	Amp
					Glucose-6-phosphate dehydrogenase	0.08	4.15	0.05	0.52	26.39	26.91	Amp
					Phosphogluconate dehydrogenase	0.08	4.15	0.05	0.52	26.39	26.91	Amp
					Homoserine dehydrogenase NADH irreversible	0.05	4.55	0.05	0.32	28.95	29.26	Amp
					Homoserine kinase	0.05	4.55	0.05	0.32	28.91	29.22	Amp
					6-phosphogluconolactonase	0.08	4.15	0.05	0.52	26.39	26.91	Amp
					Ribulose-5-phosphate-3-epimerase	0.08	4.15	0.05	0.52	26.39	26.91	Amp
					Ribose-5-phosphate isomerase	3.31	4.01	0.06	21.52	26.04	47.56	Amp
					Threonine synthase	0.05	4.55	0.05	0.32	28.91	29.22	Amp
					Transketolase	0.08	4.15	0.05	0.52	26.39	26.91	Amp
					Transketolase	0.08	4.15	0.05	0.52	26.39	26.91	Amp
6	Isobutyl alcohol	*	Succinate	0.67	Pyruvate decarboxylase	8.73	6.70	0.11	88.74	61.27	150.02	KO
**7**	L-Glutamate	*	Xanthine	*	Fructose-bisphosphate aldolase	2.81	0.95	0.05	17.90	6.04	23.94	Amp
					Glyceraldehyde-3-phosphate dehydrogenase	2.81	0.95	0.05	17.90	6.04	23.93	Amp
					Triose-phosphate isomerase	2.81	0.95	0.05	17.90	6.04	23.94	Amp
8	Sorbitol	*	L-Methionine	*	Ribose-5-phosphate isomerase	5.61	1.28	0.06	36.45	8.29	44.74	Amp
9	2,3-Butanediol	*	L-Serine	*	Fructose-bisphosphate aldolase	9.30	4.74	0.05	59.21	30.19	89.40	Amp
					Glyceraldehyde-3-phosphate dehydrogenase	9.30	4.74	0.05	59.19	30.19	89.37	Amp
					Triose-phosphate isomerase	9.30	4.74	0.05	59.21	30.19	89.40	Amp

WT, wild type; *, less than 10^−5^ mmol/gDW/h.

#### 3.2.3 Higher-Order Intervention Strategies**—**Co-Production of Isobutanol and Succinate

Higher-order intervention strategies can increase the maximum yield achievable for any product with a little difference in growth rate when compared to single interventions. But they are more cumbersome to identify, as the problem becomes time-consuming and computationally expensive. Instead of identifying higher-order targets for all metabolites in an organism, we have used the previous analysis to explore the metabolic capabilities of the organism and chose one set of metabolites to demonstrate the power of higher-order intervention strategies.

Isobutanol is a long-chain alcohol that is an attractive biofuel (Nanda et al., 2017). Succinic acid is an important metabolite essential for the production of various other products like biodegradable polymers, fatty acids, butyrolactone and tetrahydrofuran (Akhtar et al., 2014). The co-production of isobutanol and succinate has been proposed as a sustainable and economical process by Xu et al. (2018). They have discussed the development of various strains for the production of isobutanol and succinate separately. They emphasize how the co-production of isobutanol and succinate is not only of economic significance, but the high amount of carbon dioxide released from long-chain alcohol fermentation can be used for succinate production, and is hence also of ecological importance. But the article does not discuss any strategy to co-optimize the production of isobutanol and succinate.

**FIGURE 3:**
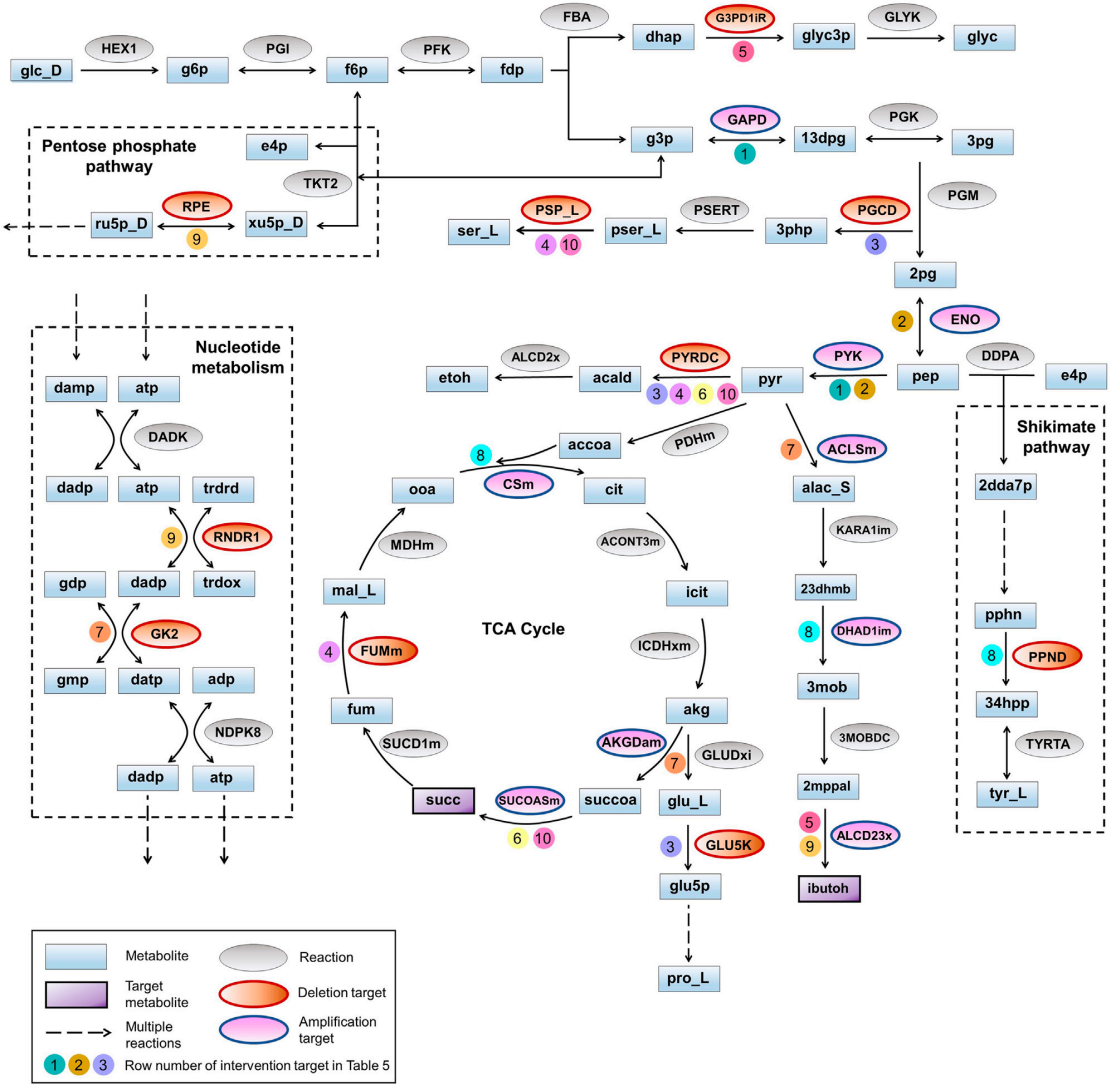
Pathway diagram for co-production of isobutanol and succinate. The pathways for production of isobutanol and succinate are represented along with the intervention strategies listed in Table 5. Auxiliary pathways that are not directly related to the biosynthesis of target products but contain intervention strategies are shown within dotted rectangles. Co-factors and other small molecules are not depicted for better clarity. Numbers on the arrows point to row numbers in Table 5.

Here we identified the higher-order intervention strategies (size up to three) for co-production of isobutanol and succinate in *S. cerevisiae* in aerobic conditions. More than 3,700 interventions can improve the yield of both the metabolites when compared to the wild-type strain. Table 5 lists a few examples of each type of intervention strategy obtained, which are also represented in Figure 3. Though most of the amplification and deletion targets are integral components of the target product and by-product synthesis respectively, co-FSEOF is also able to find intervention targets in distant pathways like those in pentose phosphate pathway, shikimate pathway, and nucleotide metabolism. This shows the ability of the algorithm to identify non-intuitive targets. Also, a number of the targets predicted here are also found in experimental studies reported in literature. For example, the deletion of pyruvate decarboxylase has been shown to improve the production of isobutanol by Kondo et al. (2012). Zahoor et al. (2019) have shown that both pyruvate decarboxylase deletion and fumarase deletion can increase the production of succinate. This shows the dependability of the results obtained using the algorithm.

**TABLE 5:** Higher-order intervention strategies for co-production of isobutanol and succinate in *S. cerevisiae* under aerobic conditions.

#	Intervention 1	Intervention 2	Intervention 3	A ⊕/K ⊝	Mutant flux 1	Mutant flux 2	Biomass flux	Score A	Score B	Score A + B
1	Glyceraldehyde-3-phosphate dehydrogenase (GAPD)	Pyruvate kinase (PYK)	NA	⊕⊕	7.19	8.60	0.20	81.73	97.87	179.59
2	Enolase (ENO)	Pyruvate kinase (PYK)	NA	⊕⊕	7.13	8.60	0.20	81.08	97.87	178.95
3	Glutamate-5-kinase (GLU5K)	Phosphoglycerate dehydrogenase (PGCD)	Pyruvate decarboxylase (PYRDC)	⊝⊝⊝	7.96	5.80	0.19	79.81	58.12	137.93
4	Fumarase (FUMm)	Phosphoserine phosphatase (PSP_L)	Pyruvate decarboxylase (PYRDC)	⊝⊝⊝	7.95	5.78	0.19	79.78	58.05	137.84
5	Aldehyde dehydrogenase (ALCD23x)	Glycerol-3-phosphate dehydrogenase (G3PD1iR)	NA	⊕⊝	2.99	2.52	0.22	43.76	36.77	80.54
6	Succinate CoA ligase ADP forming (SUCOASm)	Pyruvate decarboxylase (PYRDC)	NA	⊕⊝	7.63	5.45	0.19	79.31	56.69	136.00
7	Acetolactate synthase (ACLSm)	Oxoglutarate dehydrogenase lipoamide (AKGDam)	Guanylate kinase (GK2)	⊕⊕⊝	4.72	6.89	0.15	34.51	50.38	84.89
8	Citrate synthase (CSm)	Dihydroxy-acid dehydratase (DHAD1im)	Prephenate dehydrogenase (PPND)	⊕⊕⊝	4.73	6.98	0.14	32.31	47.71	80.02
9	Aldehyde dehydrogenase (ALCD23x)	Ribonucleoside-diphosphate reductase (RNDR1)	Ribulose 5-phosphate 3-epimerase (RPE)	⊕⊝⊝	2.99	2.48	0.22	43.84	36.32	80.16
10	Succinate CoA ligase ADP forming (SUCOASm)	Phosphoserine phosphatase (PSP_L)	Pyruvate decarboxylase (PYRDC)	⊕⊝⊝	7.95	5.78	0.19	79.78	58.05	137.84

⊕, Amplification of reaction; ⊝, Knock-out of reaction.

## 4 Discussion

Chemical processes based on fossil fuels are cheaper when compared to bioprocesses, which leads to reluctance in the adoption of sustainable bioprocesses in industries. To improve the economic feasibility of a bioprocess, we can optimize the process variables and/or genetically engineer the microbes (Dzurendova et al., 2020). Even then, in some cases, the bioprocess might be less lucrative when compared to their chemical counterparts (Zhang et al., 2021). In such cases, we can co-produce multiple metabolites to improve the economic feasibility and efficiency of a bioprocess. For example, in the case of biofuels and fatty acids, we need to design processes that can support the production of multiple metabolites of similar nature (Xin et al., 2018). Co-production also ensures better utilization of microbial capabilities, and better balance in the carbon metabolism (Fan et al., 2018). While there are multiple computational tools and algorithms to identify intervention strategies for a single product, there is a lack of readily appliable algorithms for co-production. As a result, almost no co-production study in existing literature was found to use computational algorithms to aid rational strain design. All of the studies rely on previous findings or readily apparent strategies to achieve co-production. This limits the intervention strategies designed.

In this study, we present co-FSEOF, by adapting the effective FSEOF algorithm to study the co-optimization of a set of metabolites. FSEOF is a well-established constraint-based modelling algorithm, which has been used to reliably predict metabolic engineering strategies for a variety of systems (Choi et al., 2010; Boghigian et al., 2012; Badri et al., 2019; Srinivasan et al., 2019). It has a simple and efficient framework and can identify both deletion and amplification targets. Flux Coupling Analysis (FCA) is yet another interesting algorithm that can identify which metabolites can be coupled together. But very few metabolites are innately coupled without interventions. Moreover, it excludes all the reactions that do not carry flux under a given set of conditions from the analysis. This affects the applicability of FCA because these reactions, though not coupled, can have an effect on target production in the presence of other interventions. co-FSEOF is able to identify more combinations of products that can be co-produced and also provides a wider range of intervention strategies for a given set of metabolites.

Using co-FSEOF, we examined the co-production of multiple pairs of metabolites, and both deletion and amplification targets were obtained in *E. coli* and *S. cerevisiae* under both aerobic and anaerobic conditions. Anaerobic fermentation enabled the co-production of a higher number of metabolites when compared to aerobic fermentation in both organisms. This could be due to the incomplete respiration in the absence of oxygen that leads to the formation of multiple by-products. Also, *S. cerevisiae* produces more industrially significant metabolites when compared to *E. coli.* Some of these proposed intervention strategies have been verified experimentally by other studies in literature, as mentioned in Section 3. This shows the efficacy of the algorithm in furnishing reliable targets. In addition to readily apparent intervention strategies, co-FSEOF also provides non-intuitive intervention strategies that are present in auxiliary biochemical pathways (as discussed in Section 3.2.2, 3.2.3).

The co-optimization analysis for all possible pairs of metabolites in the network is intended to be exploratory in order to give a larger picture of the metabolic capabilities of the organism. This analysis showed that around 200 pairs could be co-optimized in *E. coli* under aerobic conditions, and around 1,000 pairs of metabolites could be co-optimized in the other cases. An important class of metabolites observed in the analysis are alcohols such as ethanol, isobutanol, and 2,3-butanediol, which can be co-produced using various interventions. Co-optimization can thus enable the efficient production of biofuels (Collas et al., 2012). We can explore the metabolic capabilities of the organism to identify all possible pairs of metabolites that can be co-produced. Following this, important and commercially valuable pairs of metabolites can be further studied to obtain higher-order intervention strategies, as shown in Section 3.2.3. Exploring the higher-order strategies can expand the efficiency of the intervention targets obtained. Since the evaluation of higher-order intervention strategies is laborious and computationally expensive, we have limited the size to a maximum of three manipulations at a time. It not only enhances yield, but also provides alternate routes to achieve a similar yield. The advantageous strategies can be chosen based on the ease of manipulation in an experimental setup in such cases.

The evaluation of the results is carried out using FVA, which ensures the robustness of the targets obtained. While FBA provides one optimal solution from the solution space, FVA gives the entire range of values the flux can take up. This is a significant difference that sets co-FSEOF apart from other existing algorithms like OptKnock (Burgard et al., 2003) and OptReg (Pharkya and Maranas, 2006). Also, the algorithm validates and returns all the intervention strategies in a single run, contrary to the existing algorithms, most of which are sequential and require a separate run for each strategy obtained. The set of intervention strategies validated through FVA can be short-listed for experimental verification using the scores. The overall score  can be used to compare the effectiveness of different intervention strategies. The score is designed to incorporate both increase in product flux and decrease in biomass flux, so that both biomass and product production are favored in the mutant. Moreover, co-FSEOF uses biomass as the objective throughout the analysis, and the effect of the intervention strategies on product synthesis is studied when the organism optimizes growth. Thereby, the intervention strategies result in the co-optimization of product production and biomass formation. An intervention strategy with better overall score ensures better product synthesis along with good biomass formation. If one product is more favored than the others economically or otherwise, we can use the individual scores $Score_{i}$ to choose the appropriate strategy for the process that is formulated. The products can be chosen based on their economic value, or ease of co-production. One drawback of co-production is the cost associated with downstream processing. But this can be overcome by choosing easily separable products or choosing metabolites such that one is accumulated in the cell and one is secreted out, as in the case of polyhydroxy butyrate and succinate, respectively (Kang et al., 2010). However, this problem does not occur in the case of biofuels where the alcohol mixture is optimized for and therefore does not require extensive separation of the products. Co-FSEOF not only identifies intervention strategies for co-production of a given set of metabolites, but also allows us to explore the different combinations of products that can be co-produced in an easy and efficient manner.

## 5 Conclusion

Co-production can open new avenues for the sustainable production of chemicals. Designing bioprocesses for co-production using laboratory experiments alone is cumbersome and can result in sub-optimal strategies. co-FSEOF empowers us to explore and exploit microbial systems in a better fashion. It can be used to computationally study and optimize co-production by identifying intervention strategies for multiple metabolites and thereby improve the efficiency of bioprocesses. It should be noted that the co-optimization analysis was limited to pairs of metabolites to reduce the computational time. Nevertheless, co-FSEOF can be easily extended to co-optimize a more extensive set of metabolites. To conclude, this study can be used to identify various genetic manipulations that can co-optimize a set of products, which might be challenging to achieve through pure experimentation. It provides a novel and critical approach to study co-production computationally. We hope this study will aid the design and development of more sustainable bioprocesses.

## Data Availability

Publicly available datasets were analyzed in this study. This data can be found here: http://bigg.ucsd.edu/. All models used in this work and the codes used for our analysis are available at https://github.com/RamanLab/co-FSEOF.
